# MORE FILIAL PIETY FROM ADULT CHILDREN, MORE POSITIVE SELF-PERCEPTION OF AGING IN OLDER PARENTS?

**DOI:** 10.1093/geroni/igad104.0252

**Published:** 2023-12-21

## Abstract

Filial piety, as a central concept in Confucianism, is about the ideas of how children should treat their parents. Previous studies on filial piety have focused on older generation’s well-being. However, whether filial piety of adult children could influence their parents’ self-perception of aging on a longitudinal dyad basis is largely unknown. Drawing on the Dual Filial Piety Model and the intergenerational contact perspective, we investigated the differential impacts of authoritarian vs. reciprocal filial piety of adult children on older parents’ self-perception of aging using a longitudinal parent-child dyads dataset from a large population-based survey (i.e., Chinese Longitudinal Healthy Longevity Survey, CLHLS) from wave 2002 to wave 2005. Self-perception of aging of older parents was measured at two time points, authoritarian and reciprocal filial piety of adult children was measured at wave 2002. Latent change score modeling was performed on a 2387 parent-child dyads sample (Mage = 80.03, SD = 9.86 for parent; Mage = 48.54, SD = 8.10 for child) to test our research hypothesis. Results showed only reciprocal filial piety of adult child at wave 2002 could positively predicted their parents’ latent change score of self-perception of aging 3 years later, after controlling for potential covariates. Our study suggested that the effects of adult children’s filial piety might have different effects on parents’ aging attitude, such that a reciprocal parent-child relationship rather than child’s submission to parents could improve parents’ attitude toward their own aging.

